# Effect of luteal-phase GnRH agonist on frozen-thawed embryo transfer during artificial cycles: a randomised clinical pilot study

**DOI:** 10.3389/fendo.2023.1098576

**Published:** 2023-06-09

**Authors:** Yanghong Liu, Kaishu Huang, Cheng Chen, Li Wen, Min Lei, Yabin Guo, Bin Tang

**Affiliations:** ^1^ Reproductive Medicine Center, Third Xiangya Hospital of Central South University, Changsha, Hunan, China; ^2^ Reproductive Medicine Center, The First People’s Hospital of Changde City, Changde, Hunan, China

**Keywords:** gonadotropin-releasing hormone agonist, frozen-thawed embryo transfer, artificial cycle, luteal phase support, clinical outcomes

## Abstract

**Purpose:**

This randomised clinical pilot study evaluated the effect of the mid-luteal additional single dose of gonadotropin-releasing hormone agonist (GnRH-a) on the clinical outcome of the females subjected to artificial cycle frozen-thawed embryo transfer (AC-FET).

**Methods:**

A total of 129 females were randomised into two groups (70 in the control group and 59 in the intervention group). Both groups received standard luteal support. The intervention group was given an extra dose of 0.1 mg GnRH-a in the luteal phase. The live birth rate served as the primary endpoint. The secondary endpoints were the positivity of pregnancy tests, the clinical pregnancy rate, the miscarriage rate, the implantation rate, and the multiple pregnancy rate.

**Results:**

There were more positive pregnancy tests, clinical pregnancies, live births, and twinning pregnancies, and fewer miscarriages observed in the intervention arm compared to the controls, though no statistical significance was concluded. No difference was found in the number of macrosomia in the two groups. There was no congenital abnormality newborn.

**Conclusion:**

Overall, the difference of 12.1 percentage points in the live births rate (40.7% vs 28.6%) between the two groups, however, is statistically insignificant. the improvement of the pregnancy outcome supports the non-inferiority of GnRH-a added during the luteal phase in AC-FET. Larger-scale clinical trials are required to further establish the positive benefits.

## Introduction

With the success of improved cryopreservation techniques, pregnancy rates between fresh and frozen-thawed embryo transfer (FET) cycles are close to equal (1). It has been proposed that the ‘freeze all’ policy is challenging the current management practices in assisted reproduction treatment (ART). The artificial cycle (AC) is a prevalent option for endometrial preparation in FET, due to its clinical practicability and less demand for monitoring. Due to the lack of corpus luteum, luteal phase support (LPS) in AC-FET is essential to induce endometrial receptivity and maintain the ongoing pregnancy. Meanwhile, luteal phase defect (LPD) may result in the suboptimal responsiveness of the endometrium to progesterone” (2).

Natural progesterone is a common therapeutic agent during LPS. It can be administered in different doses and ways, alone or in combination with estradiol, human chorionic gonadotropin (HCG), or gonadotropin-releasing hormone agonist (GnRH-a) (3). The most effective LPS medication, as well as its dosage, duration, and timing of commencement and cessation, are still in dispute at the moment (4, 5). In 2004, Tesarik et al. conducted the first prospective controlled study to evaluate the effects of GnRH-a administration as luteal support on AC for fresh embryo transfers in recipients of donated oocytes (6). In recent years, GnRH-a has been utilized as a solo luteal phase support in fresh cycles (7), or as an addition to progesterone treatment in fresh cycles, natural cycle (NC), or AC FETs, in either a single or recurrent dose to treat luteal phase deficiency. There are differences in hormonal regulation and corpus luteum function in different embryo transfer protocols. The best evidence that available data regarding the beneficial effects of GnRH-a for the luteal phase on pregnancy outcome is from fresh cycles (8–12). It is still under debate if GnRH-a as an add-on to progesterone supplementation in FETs improves pregnancy outcomes. Preliminary data suggested the benefit of the administration of GnRH-a as luteal support in NC-FET (13, 14). However, the effect of GnRH-a in AC-FET remains controversial (15–18). There is a limited number of studies. The exact underlying mechanism of luteal-phase GnRH-a is still not clear, although it is hypothesized that GnRH-a enhances corpus luteum function by inducing luteinizing hormone (LH) secretion from pituitary gonadotroph cells or stimulates endometrial GnRH receptors.

This study is a prospective randomized clinical pilot trial that evaluated the efficacy and safety of the additional single dose of GnRH-a at the time of implantation on the pregnancy outcome of patients undergoing hormonally substituted AC for FET. We also investigated the effect of GnRHa administration on the birth weights of the newborns and the rate of congenital malformations because GnRH-a is hypothesized that the hormone will alter the interaction of invading trophoblast and decidua, potentially affecting placentation.

## Materials and methods

This study is a prospective clinical pilot trial with randomization, approved by the Ethics Committee of the Reproductive Center of the First Hospital of Changde City. The study complied with the principles of the Declaration of Helsinki. All patients were well-informed and the formal consent was acquired. The females were recruited between July 2018 and December 2018 from the Reproductive Center of the First Hospital of Changde City, China.

### Study population

**Figure 1:**
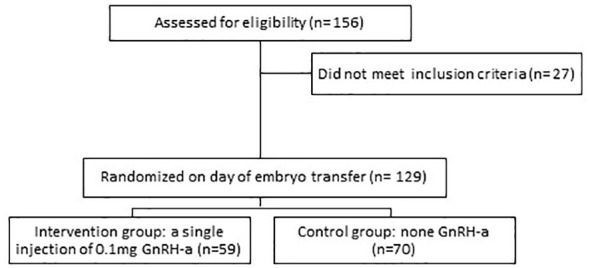
Flowchart of study design.

A total of 156 patients were recruited for AC-FET. Exclusion criteria included females over 40 years of age or follicle-stimulating hormone (FSH) ≥20IU/L, uterine anomalies, intramural myomas (≥4 cm), submucous fibroids, endometrium thickness less than 7 mm before embryo transfer, patients who had untreated systemic or endocrine disorders such as diabetes mellitus, thyroid dysfunction or hyperprolactinemia and female or male chromosomal abnormality (**Figure 1**).

### Study design

All participants were assigned to the intervention or control group according to the random number table on the day of embryo transfer. Each female was enrolled and studied only once. Patients were administered estradiol valerate ranging from 6 to 8 milligrams daily from day 2 or day 3 of the cycle, followed by daily progesterone injections (total dose 80 mg) when the endometrial thickness reached 7mm at least. The thickness of the endometrium was assessed with a transvaginal ultrasound. Transfer of embryos in the cleavage stage on the fourth day after progesterone conversion and blastocysts on the sixth day. The quality of the frozen-thawed embryos was recorded in the data. All embryos were frozen by vitrification and graded according to the Istanbul Global Consensus Scoring System (19). The percentage of fragmentation, the evenness of each blastomere, and whether multinucleation was present were assessed to grade day 3 embryos as Grade 1 to Grade 3. Good-quality day 3 embryos were defined as 7-9 cells with Grade1-2 (<25% fragmentation, equal-sized blastomeres in the majority of cells, and no multinucleation) in this study. Day 5 embryo quality was assessed based on the Gardner and Schoolcraft scoring system. The degree of expansion (Grade 1-6), inner cell mass morphology (Grade A-C), and trophectoderm morphology (Grade A-C) were used to grade day 5 embryos. Good-quality day 5 embryos were defined as Grade better than 3BB in this study. Randomization was done on the day of embryo transfer: A single dose of 0.1 mg GnRH-a (Decapepty l; Ferring, Germany) was injected subcutaneously into patients in the intervention group (n=59) when the age of the embryo was six. The patients in the control group (n=70) did not receive it. Both groups received daily progesterone injections (total dose of 80 mg) and estradiol valerate 6-8 mg. The serum β-subunit of human chorionic gonadotropin (β-hCG) concentration was used to diagnose chemical pregnancy 14 days after the day-three embryo transfer and 12 days after the day-five blastocyst transfer. The clinical pregnancy was confirmed by the detection of fetal heart activity using transvaginal ultrasound 14 days after a positive hCG test. Hormone replacement therapy was administered until either the negative pregnancy test or the tenth week of gestation.

### Outcome measures

The endpoint was live birth. The main outcome evaluations included the rates of the positive pregnancy test, the clinical pregnancy, the miscarriage rate, and the live birth rate. The rates for implantation, chemical pregnancy, and multiple pregnancies were documented. The serum β-hCG level of the patients and the weight of the newborns were recorded as well.

### Statistical analyses

The statistical analysis was performed using GraphPad, version 6.0. T-tests and Chi-square tests were used for comparing differences between categorical data. P<0.05 was considered statistically significant.

## Results

A total of 129 patients who met the inclusion criteria were enrolled (59 in the intervention group and 70 in the control group). The baseline clinical characteristics of the two study groups are shown in **Table 1**. No significant difference was observed in age, body mass index (BMI), FSH, duration of infertility, primary infertility rate, endometrial thickness at transfer day, number of transferred embryos, completely survived embryos rate, and number of good quality embryos (**Table 1**).

**Table 1:** Baseline clinical characteristics of females and embryos at the time of artificial cycle frozen embryo transfer.

Variable	Intervention group (n = 59)	Control group (n = 70)	P value
Female age at FET	30.14 ± 0.43	30.21 ± 0.42	0.897
BMI (kg/m2)	22.67 ± 0.46	23.55 ± 0.50	0.206
FSH (mIU/mL)	8.13 ± 0.34	7.36 ± 0.31	0.094
Duration of infertility (year)	3.64 ± 0.28	4.10± 0.37	0.326
Primary infertility (%)	29 (49.2)	35 (50)	0.924
Etiology for infertility (%)			0.724
Female only	50 (84.7)	59 (84.3)	
Male	4 (6.8)	3 (4.3)	
Combined	5 (8.5)	8 (11.4)	
Endometrial thickness at transfer day (mm)	9.79 ± 0.22	11.25 ± 1.51	0.343
Number of transferred embryos/FET cycle	2.02 ± 0.07	2.00 ± 0.07	0.863
Completely survived embryos (%)	124(98.4)	164(98.2)	0.750
Number of good quality embryos	1.32 ± 0.12	1.31 ± 0.11	0.962
Number of FET cycles with blastocyst (%)	3 (5.1)	5 (7.1)	0.726

Values are mean ± SD unless stated otherwise.

BMI: body mass index; FET: frozen embryo transfer.

A slightly higher β-hCG level and increased implantation rate were observed in the intervention group comparing to the controls, although the differences were not statistically significant. There was a higher rate of positive pregnancy tests in the intervention group than the rate of the control group (57.6% vs 42.9%). Correspondently, the clinical pregnancy rate was 9.2 percentage points higher in the intervention group comparing to the controls (49.2% and 40.0%); Females in the intervention group had a 12.1 percentile higher live birth rate (LBR) versus the control group (40.7% vs 28.6%). Correspondently, the miscarriage rate was 14.7 percentage points lower (10.3% vs 25%). The differences were notable in the clinic although no statistical significance was concluded. From the intervention group, 59 females gave birth to 30, while only 21 births were counted in 70 females in the control group. The number of macrosomia in both the intervention group and the control group is similar. No difference was observed in the numbers of chemical pregnancy, the ectopic pregnancy, and the twining pregnancy between the two groups (**Table 2**). All newborns were followed up for 12 months. There was no congenital abnormality newborn.

**Table 2:** Clinical outcomes of artificial cycle frozen embryo transfers receiving standard hormonal substitution and additional single dose triptorelin acetate for luteal support.

Variable	Intervention group (n = 59)	Control group (n = 70)	P value
Positive pregnancy (%)	34/59(57.6)	30/70(42.9)	0.095
β-hCG (IU/L)	1338 ± 234.8	976.5 ± 130.8	0.184
Implantation rate (%)	36/119(30.3)	31/140(22.1)	0.138
Clinical pregnancy (%)	29/59(49.2)	28/70(40)	0.374
Live birth	24/59(40.7)	20/70(28.6)	0.208
Chemical pregnancy (%)	4/34(11.8)	2/30(6.7)	0.433
Miscarriage (%)	3/29(10.3)	7/28(25)	0.269
Ectopic pregnancy (%)	2/29(6.9)	1/28(3.6)	0.975
Twinning pregnancy (%)	8/29(27.6)	3/28(10.7)	0.162
Macrosomia (%)	3/30(10)	2/21(9.5)	0.673

## Discussion

This prospective randomized clinical pilot trial investigated the effects of GnRH-a as an addition to progesterone luteal support on implantation, clinical pregnancy, and LBR. Another advantage of our study is data on the live birth and perinatal outcomes following the administration of single-dose GnRH-a in the luteal phase of AC-FET, including the rates of macrosomia and the congenital abnormalities of newborns. Recently, Ye et al. provided evidence demonstrating that GnRH-a administration in AC-FET cycles did not increase clinical or ongoing pregnancy. However, the result showed the implantation rate was significantly higher in 35~37 years old females with GnRH-a. They suggested that GnRH-a add-up could improve the implantation rate in the peri-implantation window in aging females (about 37 years old) *via* a direct effect on the embryo and enhancing embryo developmental potential (18). In our study, the intervention group had higher rates of a positive pregnancy, implantation, clinical pregnancy, and live birth. The differences range from 8.2 to 14.7 percentage points. At the same time, we found that the miscarriages were less frequent with additional GnRH-a. Despite the differences are not statistically significant. Apart from the risk of miscarriage, it should be noted that the rate of twin pregnancy in the intervention group was slightly higher than that in the control group. A safety profile regarding the effect of any add-ons used in ART on the health of newborns is crucial. Zhou et al. conducted a retrospective analysis to investigate the efficacy and safety of mid-luteal GnRH-a support. The result indicated that the GnRHa group had a slightly higher twin pregnancy rate and a significantly higher rate of premature delivery. But no evident long-term effect on the newborns (20). Therefore, mid-luteal GnRH-a administration is relatively effective and safe when precautions are taken to control the number of implanted embryos and reduce the incidence of twinning pregnancy.

GnRH-a plays a role in the treatment of LPS in fresh cycles. The recent meta-analysis, which includes 13 RCTs with 3,584 cycles, indicated that the females in IVF/ICSI (Intracytoplasmic Sperm Injection Cycles) received GnRH-a for luteal support had a significantly higher implantation rate and higher rates of pregnancy, clinical pregnancy, and live birth (21). In addition to the traditional progestogen support, a new strategy of GnRHa luteal assistance was found to improve overall IVF outcomes (22). However, with the limitations, more RCTs are required to confirm the findings (23). At present, the exact mechanism of the beneficial effects of GnRH-a in luteal phase support is still not completely understood. As novel luteal phase support, GnRH-a may act on the corpus luteum, the endometrium, and the embryo (24). GnRH-a stimulates the secretion of LH by pituitary gonadotropin cells and promotes the corpus luteum function (8). LH release stimulates angiogenetic growth factors and cytokines (25, 26). The expression of GnRH-a and its receptor were found in tissues including endometrium, ootheca, testis, placenta, and myometrium (27). Endometrium expresses GnRH and GnRH-receptor mRNA throughout all phases of the menstrual cycle, with the most intense expression during the luteal phase, and GnRH has been reported to promote adhesion between endometrial epithelial cells and the embryo (28). In human embryonic implantation, there is possibly a close interaction between the endocrine and immune systems through the GnRH and its receptor (29). Experiments *in vitro* have shown that GnRH-a can regulate the synthesis and secretion of hCG in the preimplantation embryo and placenta and improve the development of cultured embryos (30). Two studies showed that the implantation, pregnancy, and LBR increased with mid-luteal GnRH-a administration on oocyte donation (OD) cycles. The data has implied that embryo development was potentially enhanced, which might benefit from a GnRH-a direct effect on the embryo (6, 31). Based on this, the GnRH-a administration has also been adopted in FET cycles. However, oocyte donation cycles and autologous frozen embryo transfers were not identical because of a difference in the immunological milieu. Haas et al. reported that the addition of two injections of recombinant hCG and GnRH-a might increase clinical pregnancy rates on the day of transfer and 4d later, respectively, in the NC-FET (13). Seikkula et al. found a higher number of clinical pregnancies and live births in NC-FET with GnRH-a, although the statistical power was too low to show significance (14). Additionally, the lack of corpus luteum, the absence of ovulatory LH and FSH surge, and the absence of circadian oscillations of LH and FSH during the luteal phase in the NC-FET. The effect of mid-luteal GnRH-a can be different in natural and artificial FET cycles (32).

In 2015, Davar et al. designed the first prospective randomized study on GnRH-a administration in AC-FET cycles including 200 patients. On the day of the embryo transfer, the patients in the GnRH-a group were given 0.1 mg triptorelin 3 days after ET. No statistically significant difference was observed between the GnRH-a group and the controls in terms of clinical and ongoing pregnancy rates (15). While in another trial of 220 patients with AC-FET cycles, the ongoing pregnancy rate was significantly increased in the group that received GnRH-a at the time points of day 2 embryos and vitrified blastocysts (16). Seikkula et al. found that LBR was 9.8 percentage points higher in the GnRH-a group due to the lower number of miscarriages, while the clinical pregnancy rates were similar in both groups (17). The authors called for further studies to confirm the effect of GnRH-a on trophoblast–endometrial interaction. Alsbjerg et al. observed that although a difference of 14% in biochemical loss and 12% in total pregnancy loss in favor of GnRHa supplementation was seen, this did not achieve a significant difference (32). Adding one luteal dose of GnRHa may increase the live birth rate in individuals receiving the GnRHa-HRT protocol, according to a recent retrospective cohort research. The multivariate analysis revealed that luteal GnRHa administration was positively associated with ongoing pregnancy (OR 2.04, 95% CI 1.20–3.47, P = 0.008) and live birth (OR 2.03, 95% CI 1.20–3.45, P = 0.009) (33). In line with the study of Seikkula, our data showed a distinct but statistically insignificant difference in miscarriage (10.3% vs 25%) and LBR (40.7% vs 28.6%) between the patients with or without GnRH-a based on the standard luteal support. But in clinical practice, a difference of 12.1 percentage points in LBR would be relevant. GnRH-a administration in the luteal phase may enhance the endometrial receptivity and the embryo-endometrium dialogue by activating endocrine-paracrine mechanisms.

According to the previous studies, GnRHa seems not to have any benefit as an add-on in AC-FET cycles. Studies are mainly underpowered, and therefore larger studies in the future are needed. This pilot study was a prospective randomized trial with strict inclusion criteria. The sample size possibly accounted for the statistical interpretation of the results. There were few studies referenced when we planned to undertake this analysis. Limited research on the use of GnRHa administration during the luteal phase was conducted during fresh cycles. we could not conduct further larger-scale clinical trials before the efficacy and safety of the intervention were confirmed. We did not provide the power calculation because this study was designed as a pilot study. Post-study power analysis showed that the difference with 80% power in clinical pregnancy rate was statistical significance requiring a sample size of 194 patients per group. Nevertheless, our results may contribute to future meta-analyses. We are planning to carry out a further randomized controlled trial with a larger sample.

## Conclusion

Overall, the statistical insignificance of the 12.1 percentage point difference in the live birth rate (40.7% vs. 28.6%) between the GnRH-a group and the controls in AC-FET. However, the improvement of the pregnancy outcome supports the non-inferiority of GnRH-a added during the luteal phase in AC-FET. Our results offered informative references for further analysis and studies. The possible beneficial effect of GnHR-a in FET needs to be confirmed by further larger-scale clinical trials.

## Data availability statement

The original contributions presented in the study are included in the article/supplementary material. Further inquiries can be directed to the corresponding author.

## Ethics statement

The studies involving human participants were reviewed and approved by the Ethics Committee of the Reproductive Center of the First Hospital of Changde City. The patients/participants provided their written informed consent to participate in this study.

## Author contributions

YL was the principal investigator, designed the study, performed the statistical evaluations, wrote the first draft, took part in discussions regarding the results, and edited it in all its revisions. CC, LW, ML, YG, and BT participated in designing the study, edited and proofread the paper, and took part in discussions regarding the results. KH participated in designing the study, retrieved the data, assisted in writing the paper, edited and proofread the paper, and took part in discussions regarding the results. All authors contributed to the article and approved the submitted version.
